# Editorial: Challenges and successes of One Health in the context of planetary health in Latin America and the Caribbean

**DOI:** 10.3389/fpubh.2022.1081067

**Published:** 2022-12-23

**Authors:** Christina Pettan-Brewer, Daniela P. Figueroa, Natalia Cediel-Becerra, Laura H. Kahn, Andreza Francisco Martins, Alexander Welker Biondo

**Affiliations:** ^1^Department of Comparative Medicine, School of Medicine, University of Washington, Seattle, WA, United States; ^2^One Health Brasil and One Health Brazil Latin America, São Paulo, Brazil; ^3^One Health Latin America Ibero and Caribbean Network, Programa Ibero Americana de Ciencia Y Tecnologia para el Desarollo, Santiago, Chile; ^4^Facultad de Artes Liberales, Universidad Adolfo Ibáñez, Santiago, Chile; ^5^Department of Veterinary Sciences, Universidad de la Salle, Bogota, Colombia; ^6^Princeton Survey Research Center, Princeton School of Public and International Affairs, Princeton University, Princeton, NJ, United States; ^7^One Health Initiative Pro-Bono, New York, NY, United States; ^8^Department of Microbiology, Institute of Basic Health Sciences, Federal University of Rio Grande do Sul, Porto Alegre, Rio Grande do Sul, Brazil; ^9^Department of Veterinary Medicine, Purdue University, West Lafayette, IN, United States; ^10^Department of Comparative Pathobiology, Federal University of Paraná, Curitiba, Paraná, Brazil

**Keywords:** One Health (OH) approach, Latin America and Caribbean, planetary health collaborations, implementation, challenges and successes, One Health Actions, One Health (OH) history, vulnerable communities

## Introduction

One Health is currently defined, according to the 2021 advisory One Health High-Level Expert Panel, as an integrated, unifying approach aiming to maintain a sustainable balance and optimize the health of persons, animals, and ecosystems, recognizing that humans health, domestic and wildlife health, plant (authors suggest that this term should include other photosynthetic organisms such as algae and some bacteria which also play a key ecological role) health, and the wider environmental health (ecosystems) are closely integrated and interdependent (1). Such an approach mobilizes multiple sectors, disciplines, and communities at different societal levels to work together to foster wellbeing and face health and ecosystem threats while addressing community demands for clean water, energy, and air, safe and nutritious food, and integrated livestock-forest-agriculture systems, all contributing to sustainable development considering climate change evidence. On 17 March 2022, the four international agencies such as FAO, WHO, WOAH, and UNEP (One Health Quadripartite) signed a ground-breaking agreement to strengthen cooperation in a new era of One Health collaboration.^1^

Latin America faces significant challenges in the last decades due to the deep social inequality associated with environmental degradation and biodiversity loss that menace the integral health of the diverse socio-ecological systems. We consider that planetary health's (PH) core values should be addressed in this editorial as its main goal is equity in health, which is related to socioeconomic regional factors. Despite one of the main critics of PH being anthropocentric, focusing only on human health outcomes and limiting the discussions to sustainability from a human utility perspective (2), we identify in most of the articles of this special Research Topic the relevance and connection between human health disparities and worse animal health and ecosystem conservation. In addition, excessive anthropogenic activities have been leading to climate change, air, or water pollution, higher carbon emission, land degradation, and extreme deforestation. PH is crucial in Latin America and the Caribbean countries, and integrated health approaches are pivotal to mitigating climate change and should be considered as a scope of multidisciplinary collaboration under the umbrella of One Health.

One Health encompasses transdisciplinary collaborations from diverse professional backgrounds, disciplines, cultures, authorities, and community leaders for solving societal human, animal, and environmental health problems. Integrated practices are active around the world to overcome complex problems that impact the health of all living creatures, ecosystem threats, substantial biodiversity degradation, and social equity. The United Nations for the 2030 Sustainable Development Goals.^2^ aimed at a massive reduction of poverty by 2050 while maintaining environmental sustainability.^3^ Veterinarians play an important role toward these goals through their contribution to human and animal health and wellbeing, economic development, and environmental sustainability. As health professionals, we can achieve these objectives of One Health in a peaceful and sustainable environment.

## One Health challenges in the context of planetary health

Two major issues were raised with the landmark meeting of the United Nations for the 2030 agenda. First, animals were mentioned only once within the document, and only as a need for genetic diversity of domesticated animals and their related wild species. Animal health includes companion, livestock, and wildlife health and should be a full and standalone topic, as vertebrate and invertebrate animals have a crucial role in planetary health. Second, there is a practical misconception of sustainability as it does not include and/or overlap animal and plant health. According to a United Nations list of examples of sustainability indicators.^4^ used worldwide, the only three indicators of animal and/or vegetal health were biodiversity, forest area, and threatened species. The United Nations World Bank has provided a series of World Development Indicators (WDI), comprising a list of about 140 indicators for a sustainable environment of natural resources use and changes in the natural and anthropized environment. Again, such a detailed list involved the use of environmental resources, such as forest, water, cultivable land, and energy, and monitoring of environmental degradation including pollution, deforestation, and loss of habitat and biodiversity, but nothing directly regarding animal use, health, welfare, and balance of domestic, wildlife, and livestock fauna. This comprehensive list with over 40 indicators has included poverty, population stability, human health, living conditions, coastal protection, agricultural conditions, ecosystem stability, atmospheric impacts, generation, consumption, economic growth, and accessibility. Moreover, the other sustainability indicators, such as air, land, water, ecological condition, and human exposure and health, are directly related to human health and the environmental impact of anthropization.

As sustainability has been defined as the fulfillment of the current generation's demands without compromising future generations, ensuring equilibrium among economic growth, environmental care, social wellbeing, and animals were never part of the equation.^5^ Thus, a recognized prize-winning sustainable city or region may have been awarded without a single indicator of animal or plant health or wellbeing. Recently, a One Health Index (OHI) has been proposed to correct the limitations of the sustainability index based only on environmental, economic, and social overlapping domains, with few or no animal indicators inserted in the overall formula. In future, the OHI should be part of sustainability, or vice-versa, aiming for a comprehensive and extrapolable index.^6^

Finally, different understandings of the One Health concept and implementation may represent challenges, including the definition and linguistics of prevention and implementation, much to work and focus on the equal distribution of funding through the interest of individuals, institutions, and countries, overcoming the competition, and bringing more cooperation and collaboration. Meaning that even in linguistics, some say “implementation” as “initiate” while others see it as “development”—The critical part here is that they keep saying implementation in Latin America and Africa what has been implemented already and that is why new groups keep forming and starting from zero and reinventing the wheel instead of funding what has already existed—Which brings separation silos, competition, and disunion instead of the 4 Cs—Collaboration, communication, coordination, and capacity building.

## One Health demands for holistic and sustainable solutions—A global experience

Globalization, climate change, population migrations, and growing interactions among humans, animals, and plants in altered environments require that health professionals work together in a collaborative and transdisciplinary approach to improve global health and sustainability. The One Health concept encourages these collaborative partnerships, especially among health professionals from diverse interdisciplinary areas within and between countries. The goal of Global One Health is to achieve optimal global health for all species. For people, health equity and equality, gender, and race inclusiveness, especially among indigenous and minority populations, are essential. Several approaches of integrated health and “One Health” in Latin America are not new ideas, however, the concept is still being discussed, defined, and in many countries, yet to be implemented and applied. As described in this special edition by Pettan-Brewer et al., the One Health experiences in Latin America came from “*grassroots*” movements (bottom to top) through One Health Actions. Similarly, US epidemiologist Calvin Schwabe in the 20th century showed the outcomes and benefits of the “one medicine” through the interaction experiences of public health professionals and the traditional Dinka pastoral societies in 1966, reinforced in 1984, and later the evolved concept of “one health” was cited in 2002 by veterinarian Jakob Zinsstag in Chad, Central Africa, working with the integrated health nomadic pastoralists (3), although the term “One Health” was not coined until sometime between 2004 and 2007 (3).^7^

Latin American and the Caribbean public health professionals also had similar integrated human and animal health experiences in many Latin American countries. For example, the need to work as interdisciplinary teams through the simultaneous study of emerging zoonoses in people and animals, the intersectoral health economics assessment, and working together with rural and indigenous communities. *This has been the main theme of “The challenges and successes of One Health in the context of planetary health in Latin America and Caribbean” in the Frontiers Special One Health Edition*.

Historically, physicians and veterinarians worked together, but during the 20th century, they diverged. Medicine became increasingly reductionistic in its approach to diseases. Veterinary medicine and public health were relegated to second-class status. But with the challenges of the 21st century, the status quo is unsustainable. A collaborative approach became essential for individual and population health. This is the One Health-One Medicine approach, which naturally focuses on zoonotic diseases. One Health goes beyond infectious diseases. As described in this special edition, there is also the importance of governance and indigenous population participation for the success of One Health.

In contrast to other parts of the world, and similarly to Africa, One Health in most Latin America and the Caribbean countries has been practiced every day, especially in less developed and impoverished rural and urban areas where there is a lack of health professionals and a need for medical resources. The special edition included examples of the ultimate needs and examples of “grassroots” community movements working together as a necessity for education, disease prevention, and control. In other words, One Health integrated practices most likely came first, even before the actual concept was formally introduced in Latin America and the Caribbean.

## Latin America as inspirational of One Health and planetary health actions (“grassroots”)

The 2021 Frontiers Special Research Topic Edition, published in Public Health and Planetary Health journal sections, focused on One Health—*Challenges and successes of One Health in the context of planetary health in Latin America and Caribbean* included 19 articles with authors from 12 countries (Brazil, Chile, Colombia, Cuba, France, Italy, Mexico, Portugal, South Africa, Switzerland, the United States, and the United Kingdom), containing diverse topics from history, concept, implementation, research, education, and practical One Health Actions of integrated health approaches in Latin America and the Caribbean. In this scenario, the special edition has fully accomplished its main objective: a deep theoretical and practical analysis of Latin American issues and ideas on the One Health approach. Studies included owner-dog leptospirosis, malaria vectors, modern planetary health, and One Health of peripheries, unified health system, rabies in the Amazon, veterinary rescue team, biodiversity in higher education, food safety of animal origin, public policies, avian influenza virus among Cuban hunters, malaria, canine olfactory detection of SARS-CoV-2, mcr-1 gene, West Nile virus surveillance, historical concept, implementation and approach of One Health in Latin America and the Caribbean countries, social sciences in dam rupture as the worst environmental disaster, antimicrobial resistance, cross-sectoral collaborations, integrated systems for holistic approaches, indigenous health, and needs for curricular changes in primary and secondary education discussions. In addition, out of the 19 accepted peer-reviewed articles, the special edition had two studies from Guadeloupe—A French overseas region in the Caribbean Sea—A cross-sectoral collaboration to inform the implementation of the One Health approach, and an international One Health collaboration of France and Institute Pasteur of Guadaloupe conducting West Nile Virus (WNV) surveillance in a small island state of the Caribbean.^8^

Even if different sectors work differently, the cultures are different, and the approaches are different, ultimately the goal is the same. We have a lot to contribute to One Health. We need to organize and integrate our work—both ways are important: “top to bottom and bottom to top”. That is the first message. In this special edition, authors shared experiences in Latin America and the Caribbean from both sides. A second important message is that we have tools—some of which are legally binding, like International Health Organizations, that can help us and we need to rely on them. Because one of the problems we have had for decades is that with the One Health approach, everyone agrees on the principle and concept, but when it comes to implementing them, there is no necessary political will or the financial capacity to develop a One Health unit or intersectoral coordination. Regulations are used as binding instruments and it is much more likely that a One Health Agenda for good governance will be endorsed and used among agricultural, health, and all ministries. It is essential to also have these institutional tools and many times even legal dimensions. On 17 October 2022, at the Global Health Summit, in Berlin, Germany, the quadripartite (Geneva, Nairobi, Paris, and Rome) launched the One Health Joint Plan of Action (2022–2026).^9^

## Final remarks and perspectives

A challenge for One Health, and finally with the United Nations Environment Programme joining the tripartite, now known as the quadripartite, is to always include environmental health. Hence, the One Health Joint Plan of Action (see text footnote 9), developed through a participatory process of these four global organizations, provides a set of activities that aim to strengthen collaboration, communication, capacity building, and coordination across all sectors responsible for addressing health concerns at the human-animal-plant-environment interface.

1. The One Health Actions experience in Latin America is an example of bottom-up implementations similar to “grassroots” movements as described in many articles of this special edition and well-elucidated in the article by Pettan-Brewer et al. Unfortunately, even though One Health has been successfully implemented in many Latin American and the Caribbean countries for over 10 years, world recognition and funding is yet not available to continue these projects and initiatives. We hope now that with national and international One Health public policies following the Quadripartite Plan of Action (2022–2026), this important issue will be finally resolved, hence the importance of the Top to Bottom One Health implementation as well.
2. Newer associations and Latin American leaders and co-leader representatives have been very active in sharing their experiences in One Health world organizations: OHLLEP, CABI One Health Publishing, current Frontiers One Health Special Research Topics, International Alliance against Health Risks in Wildlife Trade, and Country Senators and Ministries becoming involved developing public policies and recognitions, World Health Summit 2022, United Nations Nature for Health Expert Advisor, and One Oceans Health (Beyond One Health and One Ocean Kiel Initiative.^10^)
3. Language, cultural, and political differences and lack of financial support remain a barrier and a limitation to disseminating and developing the One Health, EcoHealth, and Planetary Health concepts, and integrated health approaches in Latin America and the Caribbean countries.

Furthermore, the scope of the special One Health expert's peer-reviewed collection was achieved beyond expectations inspiring many other special topics in One Health afterward. Pioneering is only for the courageous ones—It takes determination to go through difficult adversity, criticism, and controversial discussion, and yet continue to focus on the final proposal—To transcend cultural and political differences, languages barrier, economic difficulties for research projects and publications, competitiveness, and finally achieve collaborations, partnerships, and inspire transformation. One Health will only succeed if we as humans change ourselves as individuals first, and then we can change humanity to be successful in the One Health implementation and development—From *Ego to Eco*.

In conclusion, this special Research Topic provided multidisciplinary investigations and transdisciplinary Planetary Health and One Health Actions, focusing on relevant topics for Latin America and the Caribbean countries and emphasizing transdisciplinarity in research, outreach, and education. Nevertheless, several challenges remain to be fully addressed and require more attention, particularly the impact of climate change, deforestation, the inclusion of indigenous people's knowledge, and planetary health. Sharing One Health in Latin America and the Caribbean is essential for implementing scientific priorities, supporting national and international public policies, and effective customized decision-making for each and all involved countries. We must recognize Latin American and the Caribbean knowledge, unify One Health experiences, and fortify inclusion and diversity in the One Health Global Leadership.

## Author contributions

All authors listed have made a substantial, direct, and intellectual contribution to the work and approved it for publication.

## Footnotes

^1^Available online at: https://www.who.int/news/item/29-04-2022-quadripartite-memorandum-of-understanding-(mou)-signed-for-a-new-era-of-one-health-collaboration (accessed October 25, 2022).

^2^Available online at: https://documents-dds-ny.un.org/doc/UNDOC/GEN/N15/291/89/PDF/N1529189.pdf?OpenElement

^3^Available online at: https://sdgs.un.org/2030agenda (accessed October 25, 2022).

^4^Available online at: http://wdi.worldbank.org/tables (accessed November 12, 2022)

^5^Available online at: https://www.un.org/en/academic-impact/sustainability (accessed November 12, 2022).

^6^Available online at: https://news.un.org/en/story/2016/03/524202-un-statistical-body-agrees-global-indicators-measure-sustainable-development (accessed October 25, 2022).

^7^Available online at: https://onehealthinitiative.com/history-of-the-one-health-initiative-team-and-website (accessed November 12, 2022).

^8^Available online at: https://www.frontiersin.org/research-topics/13053/challenges-and-successes-of-one-health-in-the-context-of-planetary-health-in-latin-america-and-the-c (accessed October 25, 2022).

^9^Available online at: https://www.who.int/news-room/events/detail/2022/10/18/default-calendar/one-health-joint-plan-of-action-(oh-jpa)-hybrid-high-level-advocacy-event (accessed October 25, 2022).

^10^Available online at: https://forum.oceandecade.org/events/84158 (accessed November 12, 2022).
